# Duration of hospital participation in a nationwide stroke registry is associated with improved quality of care

**DOI:** 10.1186/1471-2377-6-20

**Published:** 2006-06-01

**Authors:** Nancy K Hills, S Claiborne Johnston

**Affiliations:** 1 Department of Neurology, Box 0114, University of California San Francisco, 505 Parnassus Ave., M-798, San Francisco, CA, 94143-0114, USA; 2 Department of Epidemiology and Biostatistics, University of California San Francisco, San Francisco, CA, USA

## Abstract

**Background:**

There are several proven therapies for patients with ischemic stroke or transient ischemic attack (TIA), including prophylaxis of deep venous thrombosis (DVT) and initiation of antithrombotic medications within 48 h and at discharge. Stroke registries have been promoted as a means of increasing use of such interventions, which are currently underutilized.

**Methods:**

From 1999 through 2003, 86 U.S. hospitals participated in Ethos, a voluntary web-based acute stroke treatment registry. Detailed data were collected on all patients admitted with a diagnosis of TIA or ischemic stroke. Rates of optimal treatment (defined as either receipt or a valid contraindication) were examined within each hospital as a function of its length of time in registry. Generalized estimating equations were used to adjust for patient and hospital characteristics.

**Results:**

A total of 16,301 patients were discharged with a diagnosis of stroke or TIA from 50 hospitals that participated for more than 1 year. Rates of optimal treatment during the first 3 months of participation were as follows: 92.5% for antithrombotic medication within 48 h, 84.6% for antithrombotic medications at discharge, and 77.1% for DVT prophylaxis. Rates for all treatments improved with duration of participation in the registry (p < 0.05), with the most dramatic improvements in the first year.

**Conclusion:**

In a large cohort of patients with stroke or TIA, three targeted quality-improvement measures improved among hospitals participating in a disease-specific registry. Although the changes could be attributed to interventions other than the registry, these findings demonstrate the potential for hospital-level interventions to improve care for patients with stroke and TIA.

## Background

There are several therapies for patients with ischemic stroke that have been proven in clinical trials and are recommended widely in consensus guidelines [1-3]. These include prophylaxis for deep venous thrombosis (DVT) [4], antithrombotic therapy within 48 hours [5], and antithrombotic therapy at discharge [6]. Nationally representative data from a sample of patients discharged with ischemic stroke who utilized Medicare benefits demonstrated underutilization of antithrombotic medications at discharge, with 17% without a contraindication failing to receive them [7]. Nationally representative data on use of antithrombotic medications within 48 hours of admission and on use of DVT prophylaxis are lacking and would be useful as benchmarks for quality-improvement initiatives.

Registries have been used as tools to define deficiencies and improve quality of care. They are meant to enlighten practitioners and administrators about deficiencies, motivate change, and direct efforts [8]. When used as part of a continuous quality improvement program that includes institutional and practitioner-level feedback, registries have been demonstrated to improve care [9].

Several organizations have promoted formation of stroke registries to improve quality of care [10-13], and the Joint Commission on the Accreditation of Healthcare Organizations (JCAHO) requires maintenance of a registry to track quality-of-care indicators as part of its added certification for primary stroke centers [14]. We previously showed that quality of care improved for patients with stroke treated at six hospitals in California that implemented standardized stroke orders and tracked processes of care in a registry [15]. Using a national acute stroke registry, we sought to establish benchmarks for three quality improvement indicators recommended by the JCAHO – DVT prophylaxis and prescription for antithrombotic medications within 48 hours of admission and at hospital discharge – among patients with ischemic stroke or TIA admitted to a large sample of hospitals in the U.S. We also tested the hypothesis that duration of participation in the registry was associated with improvement in quality of care.

## Methods

A web-based acute stroke treatment registry was created as a tool for tracking hospital outcomes and improving quality of care (Ethos, The Stroke Group, Littleton, CO) [16]. The system allows hospital staff to capture, analyze, and track information on care of patients seen with stroke and TIA, and to compare results with those of other institutions in the registry. Fifty standardized data elements document aspects of acute stroke treatment, including timing of symptom onset and arrival, diagnostic evaluation, use of standard treatments (e.g., thrombolysis, antiplatelet agents, anticoagulation) during hospitalization and at discharge, documented contraindications to treatment, and discharge disposition. Staff at each hospital can access and update its own data at any time, and may also view aggregated data from all other participating hospitals on a local, state, or national level. As such, Ethos was designed to provide data acquisition and aggregation on a large scale and supports rapid-cycle performance improvement [9].

### Participating hospitals

**Figure 1:**
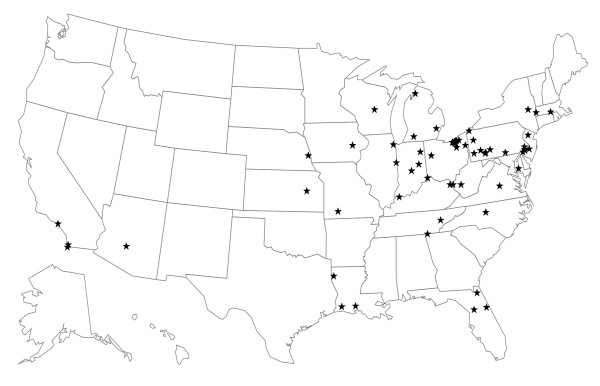
Distribution of the 86 hospitals participating in the registry. Because of proximity, not all hospitals are visible as separate stars.

Participation in Ethos is entirely voluntary and requires payment to underwrite development and maintenance costs. Eighty-six hospitals across the U.S. contributed to Ethos during the study period (Figure 1). Participating hospitals signed a contract agreeing to include all stroke and TIA admissions in the registry throughout their period of enrollment. Academic hospitals comprised 49%. Hospital catchment areas had a median population of 393,496 (intra-quartile range [IQR], 223,462–799,215). The median number of hospital beds was 326 (IQR 204–478) with a median of 275 (IQR 185–407) stroke patients treated per year. The majority of hospitals reported having standardized guidelines for stroke treatment (98%), lab support (100%), capability of treating acute stroke patients at all times (99%), and availability of a neurosurgeon within 2 hours (95%). Fewer (70%) reported the in-hospital presence of a designated stroke team.

### Data analysis

To determine whether on-going participation in the registry was associated with improvement in care, we chose to evaluate duration of a hospital's participation as a predictor of 1) the receipt of antithrombotic medications within 48 hours of ED arrival, 2) prophylaxis for deep venous thrombosis (DVT), and 3) usage of antithrombotic medications in appropriate candidates at hospital discharge. All hospitals that remained in the Ethos registry >1 year and contributed at least 50 patients with ischemic stroke or TIA were included in these analyses. Duration in registry was measured in months. To examine the receipt of antithrombotic medications within 48 hours of ED arrival and DVT prophylaxis, analyses first were performed on all patients. Analyses then were repeated on the group of patients who were discharged alive, to account for that fact that some of these patients might have died before receiving antithrombotics or DVT prophylaxis. To examine usage of antithrombotic medications at hospital discharge, we included only those patients who survived to discharge. Analyses for which DVT prophylaxis was an outcome included only ischemic stroke patients, since TIA patients do not generally require treatment.

Optimal treatment with antithrombotic medication within the first 48 hours of hospitalization was defined as having received thrombolysis (intravenous or intra-arterial tPA or an experimental thrombolytic), an antiplatelet agent (defined as aspirin, clopidogrel, ticlopidine, dipyridamole, or extended-release dipyridamole/aspirin), or an anticoagulant (warfarin or unfractionated or low-molecular-weight heparin), or having a valid contraindication to antithrombotic treatment (that is, bleeding or terminal illness, as determined by documentation in the medical records). Optimal DVT prophylaxis was defined as treatment (compression boots or anticoagulation) within 48 hours or documentation that treatment was unnecessary (such as, if the patient was ambulatory). Treatment was assessed only in patients diagnosed with ischemic stroke, since DVT prophylaxis is generally not necessary in patients diagnosed with TIA.

Optimal treatment with antithrombotic medication at discharge was defined as having received either an antiplatelet agent or anticoagulation, or having a documented valid contraindication to antithrombotic treatment. Allowable contraindications included transfer to another facility (assuming that treatment would be prescribed at the receiving facility), refusal of treatment, discharge against medical advice, allergy, bleeding risk, terminal care or illness, brain or metastatic cancer, planned surgery, or aortic dissection. Analyses using "actual treatment" as an outcome classified as treated only those patients who actually received a given medication/treatment. Therefore, both contraindicated patients as well as those without contraindications were categorized as untreated.

The data used in these analyses were collected between December 1999 and December 2003. During this period, hospitals entered and exited the registry at irregular times, and remained in the registry for widely varying lengths of time. Our primary predictor was time in registry, and time zero was defined as the date of first patient entry for each hospital. All three optimal treatment outcomes were examined within each hospital as a function of its length of time in the registry. Multivariable analyses adjusted duration in registry by type of facility (academic vs. non-academic, availability of stroke team) and patient characteristics (age, sex, race/ethnicity, diagnosis, and discharge disposition). Generalized estimating equations were used in both univariate and multivariable analyses in order to adjust for inter-hospital differences and account for clustering of observations at hospitals. Multivariable models with adjustment for calendar year (with the year 2000 as a reference) were also examined in sensitivity analysis. All analyses were performed using SAS (version 8e, SAS Institute, Cary, NC).

## Results

A total of 22,264 patients from 86 hospitals were entered into the registry. Patients from 27 hospitals that had participated in the registry <1 year (n = 2,230) and from nine hospitals that contributed fewer than 50 patients with stroke or TIA (n = 413) were excluded. Of the remaining 19,621 patients, 12,263 (62.5%) were diagnosed at discharge with ischemic stroke, 4,038 (20.6%) with TIA, 1,717 (8.7%) with intracerebral hemorrhage, 414 (2.1%) with subarachnoid hemorrhage, and 1,189 (6.1%) with conditions other than stroke. Thus, 50 eligible hospitals contributed 16,301 patients, of whom 12,263 (75.2%) were diagnosed with ischemic stroke and 4038 (24.8%) with TIA. Of these patients, 774 (4.7%) died before discharge from the hospital and were excluded from analyses for which receipt of antithrombotic medication at discharge was the outcome. For receipt of DVT prophylaxis and antithrombotics within 48 hours, no differences were observed between analyses that were inclusive or exclusive of patients who died in hospital. Results are therefore presented for analyses including all patients. The median duration of participation in the registry for these hospitals was 23 months (IQR 16–30). Age, gender, and discharge disposition were similar across years of registry participation. Although academic medical centers contributed more patients in later years (Table 1), there was no significant difference in the median time spent in registry between the two groups (22 months for non-academic hospitals compared to 24 months for academic hospitals, p = 0.47).

### Receipt of antithrombotic medication within 48 hours of arrival

**Figure 2:**
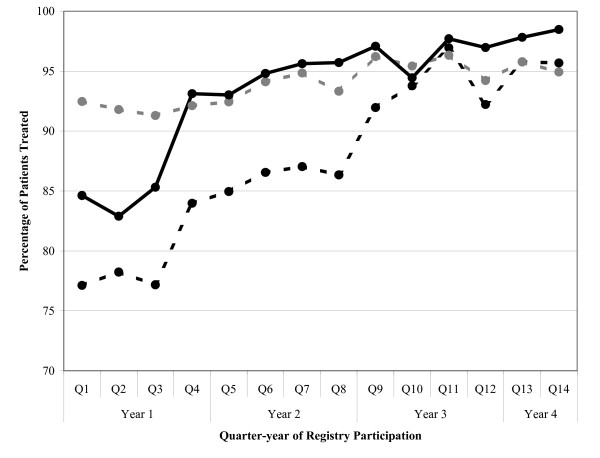
Percentage of patients optimally treated with antithrombotic medications within 48 h (gray hashed line), DVT prophylaxis (black hashed line), or antithrombotic medications at discharge (black solid line) by quarter of participation in the registry. Adherence to all three quality-improvement indicators improved with duration of participation in the registry.

Overall, 15,181 patients (93.1%) were optimally treated with antithrombotic medications within 48 h of hospital admission. The majority of these (91.8% of all patients) received antithrombotic medication or thrombolysis. Valid contraindications (primarily related to risk of bleeding or terminal illness) were cited for an additional 1.4%, and 6.8% neither received nor had a documented contraindication to antithrombotic treatment. In the first quartile of participation 92.5% were optimally treated. Longer time spent in registry was associated with increased rates of in-hospital antithrombotic use (Figure 2; Table 2). Time in registry continued to be significantly associated with rates of treatment even when adjusted for calendar year (p = 0.0005, results not shown). In an analysis ignoring contraindications, actual treatment also improved with duration of participation (Table 2).

### DVT prophylaxis within 48 hours of arrival

Because DVT prophylaxis is generally not necessary in patients diagnosed with TIA, only the 12,263 patients diagnosed with ischemic stroke were included in analyses using optimal treatment for DVT as an outcome. During the period of observation, 10,067 (82.1%) were optimally treated: 5,623 patients (45.9% of all 12,263 patients) who received DVT prophylaxis, and 4,444 (36.2% of all patients) who were documented as not requiring treatment. Thus, 2196 (17.9%) were not treated and had no documented reason for this. In the first three months of registry participation, only 77.1% were optimally treated. Increased duration of participation in registry was associated with higher rates of optimal treatment (Figure 2; Table 2). In a separate multivariable model including calendar year, duration in registry was marginally significantly associated with increased optimal treatment rates (p = 0.06, results not shown). There was no change in actual receipt of DVT prophylaxis (Table 2), so improvement was likely related to either increased early ambulation or improved documentation.

### Antithrombotic use at discharge

Overall, 14,151 patients (91.1% of the 15,527 patients who survived to discharge) were optimally treated with antithrombotic medications at discharge, either receiving them (86.8% of 15,527 patients) or having a valid contraindication (4.4%). A total of 1,376 patients (8.9%) did not receive any antithrombotic medication, and had no documented reason for non-treatment. Duration of registry participation was associated with the increased use, increasing steadily from 84.6% in the first three months of participation (Figure 2; Table 2). This association even persisted in multivariable analysis that adjusted for calendar year (p = 0.01). Ignoring contraindications, actual treatment also increased with duration of participation (Table 2).

Because the hospitals that remained in the registry the longest period of time were predominantly academic and this could bias trends, additional analyses were performed excluding academic hospitals. No significant changes in the results were observed (data not shown).

## Discussion

Among a large sample of hospitals from across the U.S., three evaluated measures of quality of care for ischemic stroke and TIA all indicated suboptimal treatment initially, but improved after implementation of a web-based registry. Improvements began in the first year but continued with longer participation (Figure 2). For use of antithrombotic medications within 48 h and at discharge, improvements were due to increases in use and were independent of calendar year, suggesting that they were not attributable to broader secular trends in usage but truly associated with registry participation. For DVT prophylaxis, improvements in optimal treatment measures could not be attributed to actual treatment and may have been due to better documentation or early ambulation. Although changes in DVT prophylaxis were dramatic in univariate analysis (Figure 2; Table 2), there were major differences between hospitals and the association was weaker after adjustment and insignificant when calendar year was included in the model.

One of the major strengths of our study was our ability to measure change over time within individual hospitals, rather than relying on aggregated data at different points in time as previous studies have done. However, given that hospitals voluntarily participating in the registry were likely to be interested in improving the quality of stroke care, our results may have been influenced by selection bias. Although the registry was likely to have played an important role in quality-improvement efforts, we have no systematic knowledge of, and therefore could not adjust for, additional hospital-level interventions that might have contributed to improvement. In fact, other studies have shown that individualized feedback is necessary to produce a substantial impact from registry data [17,18]. Thus, it is impossible to attribute the improvements in care solely to registry participation as opposed to other simultaneous interventions. Nonetheless, our study demonstrates that it is feasible to significantly improve stroke treatment over a relatively short period of time.

There are several other limitations to our study. By the very nature of registry participation, we have no control hospitals. Comparison of these results with data collection from a sample of hospitals with personnel unaware that care is being monitored would provide stronger support for the impact of the registry but may be very difficult to generate. Also, broader national trends in stroke care may have influenced outcomes regardless of registry participation. However, improvements with antithrombotic use were not explained by secular trends since they persisted after adjustment for calendar year. A randomized trial of registry implementation with continuous feedback to hospitals and physicians would provide the only incontrovertible data of efficacy. Given the potential impact of registries in improving adherence to best practices and, thus, reducing the risk of in-hospital complications and recurrent stroke, such a trial is clearly justified from a public health perspective.

Of the 50 hospitals included in these analyses, half were academic institutions. While these hospitals made up the bulk of the cohort in the last year of data collection, there was no significant difference overall in the length of time that academic hospitals participated in the registry compared to non-academic hospitals, and results did not change appreciably when analyses were performed on non-academic hospitals only. In addition, while hospitals signed a contract agreeing to enroll all stroke and TIA admissions during the duration of their participation, no system was in place to monitor whether or not this did, in fact, occur. Selective entry of patients into the registry potentially could have biased the results of the study. Another shortcoming of our study was a lack of knowledge as to why hospitals chose to withdraw from the registry once enrolled, or what factors might have contributed to continued participation over time. Data from the final year analyzed must be interpreted with caution, given that only a small number of hospitals remained in the registry for this period, and these hospitals may represent a biased sample of those institutions most dedicated to quality improvement. Lastly, the median catchment area for the included hospitals is in excess of 330,000, and results therefore may not be generalizable to smaller hospitals in rural areas. 

## Conclusion

Although voluntary disease registries have limitations due to sampling and limited oversight of data quality, they have been a major source of important health services and outcomes research in other disease areas. For example, The National Registry of Myocardial Infarction, a large voluntary web-based registry for acute myocardial infarction, has been the source of data for more than 30 major publications [19]. It has also been a source of data upon which hospitals have based quality-improvement interventions [20]. Other voluntary registries in diverse disease areas have also been the source of important research and the basis of quality-improvement interventions [21,22].

Ethos and "Get with the Guidelines" [12], a similar voluntary registry, may provide such support for those studying or caring for patients with stroke. Using data from Ethos, we have shown that, in a large cohort of patients with stroke or TIA, three targeted quality-improvement measures improved among participating hospitals. Although the changes could be attributed to interventions other than the registry, these findings demonstrate the potential for hospital-level interventions to improve care for patients with stroke and TIA. Stroke registry use is likely to increase rapidly since accreditation of hospitals as primary stroke centers by the Joint Commission on Accreditation of Healthcare Organizations requires quality-of-care measurements that will necessitate systematic collection of more detailed data [14]. Our study suggests that participation in such a registry, along with complementary hospital-wide interventions, may encourage improvement in quality of stroke care, and our baseline data, which are consistent with other studies [7], suggest that such improvement is necessary.

## Competing interests

This study was proposed and designed, and all analyses were performed and manuscript prepared independent of the sponsor, who had no control of its content.

## Authors' contributions

NH participated in the design of the study, performed the statistical analysis, and drafted the manuscript. SCJ conceived of the study, participated in its design, and helped to draft the manuscript. Both authors read and approved the final manuscript.

## Pre-publication history

The pre-publication history for this paper can be accessed here:



## Figures and Tables

**Table 1:** Demographic Characteristics by Hospital Years in Registry

Characteristic	Total (n = 16301)	Year 1 (n = 7879)	Year 2 (n = 5734)	Year 3 (n = 2313)	Year 4 (n = 375)
Age, mean(sd)	71.8 (13.6)	72.1 (13.5)	71.8 (13.6)	71.2 (13.6)	72.0 (13.5)
Female, N(%)	9071 (55.7)	4390 (55.7)	3200 (55.8)	1271 (55.0)	210 (56.0)
Ethnicity, N(%)					
Non-Hispanic white	13475 (82.7)	6369 (80.8)	4930 (86.0)	1862 (80.5)	314 (83.7)
African-American	2201 (13.5)	1163 (14.8)	611 (10.7)	372 (16.1)	55 (14.7)
Asian/Pacific Islander	113 (0.7)	74 (0.9)	26 (0.5)	10 (0.4)	3 (0.8)
Hispanic	123 (0.8)	68 (0.9)	30 (0.5)	25 (1.1)	0 0.0
Other/unknown	389 (2.4)	205 (2.6)	137 (2.4)	44 (1.9)	3 (0.8)
Academic hospital, N(%)	10255 (62.9)	4472 (56.8)	3579 (62.4)	1829 (79.1)	375 (100.0)
Ischemic stroke, N(%)	12263 (75.2)	6028 (76.5)	4229 (73.8)	1722 (74.5)	284 (75.7)
Discharged home, N(%)	8397 (51.5)	3884 (49.3)	2990 (52.2)	1307 (56.5)	216 (57.6)

**Table 2:** Duration of Registry Participation as a Predictor of Care

	Univariate Analysis					Multivariable Analysis‡		
		
Quality Measure	Year 1 (n = 7879) N (%)	Year 2 (n = 5734) N (%)	Year 3 (n = 2313) N (%)	Year 4 (n = 375) N (%)	P value*	OR§	95% CI	P value*
Optimal Treatment (those who received therapy or had valid contraindication)								
Antithrombotic therapy within 48 hours	7244 (91.9)	5365 (93.6)	2214 (95.7)	358 (95.5)	0.001	1.11	(1.04–1.20)	0.003
DVT prophylaxis within 48 hours†	4607 (76.4)	3595 (85.0)	1596 (92.7)	269 (94.7)	0.01	1.19	(1.02–1.40)	0.03
Antithrombotic medication at discharge**	6502 (86.8)	5146 (94.5)	2148 (96.5)	355 (98.1)	<0.0001	1.46	(1.24–1.71)	<0.0001

Actual Treatment (those who actually received therapy)								
Antithrombotic therapy within 48 hours	7154 (90.8)	5284 (92.2)	2174 (94.0)	349 (93.1)	0.03	1.06	(0.99–1.14)	0.08
DVT prophylaxis within 48 hours†	2516 (41.7)	2015 (47.7)	923 (53.6)	169 (59.5)	0.85	0.94	(0.75–1.18)	0.62
Antithrombotic medication at discharge**	6236 (83.2)	4877 (89.6)	2026 (91.0)	332 (91.7)	0.003	1.20	(1.06–1.36)	0.005

* Both univariate and multivariate analyses were performed using GEE to adjust for hospital differences

** Excludes patients who died in-hospital (n = 774)

† Analyses included only ischemic stroke patients (n = 12,263)

‡ Adjusted for age, gender, race, type of facility, diagnosis, presence of stroke team, discharge disposition

§Change in outcome for each 6-months in registry
